# Low-Dimensional Nanomaterial Systems Formed by IVA Group Elements Allow Energy Conversion Materials to Flourish

**DOI:** 10.3390/nano12152521

**Published:** 2022-07-22

**Authors:** Dan Li, Jinsheng Lv, Mengfan Shi, Liru Wang, Tian Yang, Ya’nan Yang, Nan Chen

**Affiliations:** 1 Key Laboratory of Cluster Science, Ministry of Education of China, Key Laboratory of Photoelectronic/Electrophotonic Conversion Materials, School of Chemistry and Chemical Engineering, Beijing Institute of Technology, Beijing 100081, China; 3120211239@bit.edu.cn (D.L.); ljs4857@163.com (J.L.); shimengfansmf@163.com (M.S.); example_wlr@163.com (L.W.); 3120201317@bit.edu.cn (T.Y.); yangyanan310@163.com (Y.Y.); 2 Yangtze Delta Region Academy of Beijing Institute of Technology, Jiaxing 314019, China

**Keywords:** low-dimensional nanomaterials, IVA group elements, battery, transducer, water evaporation

## Abstract

In response to the exhaustion of traditional energy, green and efficient energy conversion has attracted growing attention. The IVA group elements, especially carbon, are widely distributed and stable in the earth’s crust, and have received a lot of attention from scientists. The low-dimensional structures composed of IVA group elements have special energy band structure and electrical properties, which allow them to show more excellent performance in the fields of energy conversion. In recent years, the diversification of synthesis and optimization of properties of IVA group elements low-dimensional nanomaterials (IVA-LD) contributed to the flourishing development of related fields. This paper reviews the properties and synthesis methods of IVA-LD for energy conversion devices, as well as their current applications in major fields such as ion battery, moisture electricity generation, and solar-driven evaporation. Finally, the prospects and challenges faced by the IVA-LD in the field of energy conversion are discussed.

## 1. Introduction

As a guarantee for the rapid development of modern society, the important role of energy in all walks of life cannot be overstated. However, the energy crisis caused by the depletion of traditional energy sources and the environmental pollution caused by the improper use of energy are becoming increasingly prominent. Therefore, in order to tackle the global problem, an environmentally friendly and sustainable energy-saving conversion system is urgently needed [1,2,3]. The IVA group elements, represented by carbon, are widely distributed and stable in the earth’s crust and have received a great deal of attention from scientists [4,5]. The combination of the IVA group elements and nanotechnology has expanded their application. Fadaly et al. have broken through the limitation of silicon technology requiring use in conjunction with direct band gap light-emitting devices by synthesizing silicon and germanium alloy to achieve direct band gap high luminescence, enabling the integration of electronic and photoelectric functions on a chip [6]. More importantly, the unique electronic and energy band structures [7,8,9] of the IVA group elements make them widely available for efficient energy conversion and storage. Graphene, for example, is a zero-band semiconductor material with π (p) bonds, where the strength of the bonded electrons is not sufficient for these electrons to leap from p to p*. The abundant conjugated π (p) bonds promote the excited electrons at almost all wavelengths of sunlight, giving the materials a black color. The excited electrons jump from ground state orbital (HOMO) to high energy orbitals (LUMO) and then jump back to the ground state via electron-phonon coupling, releasing energy and causing lattice vibrations that lead to an increase in temperature, enabling efficient solar-thermal energy conversion.

With the abundance of methods for the preparation of low-dimensional nanomaterials, better morphology control and modification of loading groups can be achieved, enabling better applications for energy storage and conversion, and showing superior performance. The synthesis of nanomaterials is mainly divided into top-down methods, such as stripping [10], etching and laser, etc., and bottom-up methods, such as in-situ growth, hydrothermal synthesis self-assembly, etc. Li et al. used the concept of “Phoenix Nirvana” to synthesize graphene, through reconstituted graphene nanoparticles to obtain a new structure of three-dimensional (3D) graphene; the obtained porosity, electrical conductivity, mechanical strength, etc., increased greatly [11]. Stetson et al. found that the formation mechanism of the initial solid electrolyte mesophase on the silicon wafers with natural oxide and chemically etched thermal oxide coating is different, and the structural reversal of SEI is achieved by chemical etching, which can be used to improve the service life of anode materials [12]. Zhang et al. tuned the electronic properties of reduced graphene oxide (rGO) by TI atoms, and the Fermi level drop significantly reduced the series connection of carbon-based electrodes resistance, thus greatly improving the power conversion rate of C-PSC [13].

This review first describes the different morphologies and properties of IVA-LD, followed by an overview of the synthesis methods of these typical low-dimensional nanomaterials. Finally, the excellent applications and advances in energy conversion of the low-dimensional nanomaterials are presented.

## 2. Morphology and Properties of IVA-LD

### 2.1. 0D Quantum Dots

**Figure 1:**
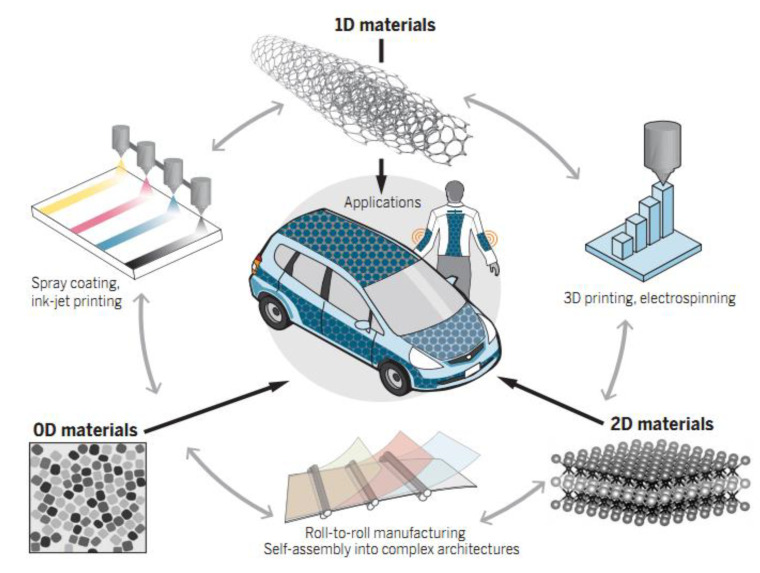
IVA-LD are widely used in energy storage and conversion devices. Reprinted with permission from Ref. [14]. Copyright 2019 American Association for the Science.

Nanostructures in different dimensions, such as quantum dots, nanotubes, nanorods/wires, and nanosheets, have provided satisfactory solutions for the rapid development of energy storage and conversion devices, as shown in Figure 1.

Quantum dots are zero-dimensional (0D) semiconductor particles, only a few nanometers in size, sometimes referred to as atoms. Like a naturally occurring atom or molecule, it has bound discrete electron states. As a carbon nanomaterial, 0D carbon quantum dots (CQDs), have attracted increasing attention in recent years because of their low cost, non-toxicity, large surface area, high electrical conductivity, and abundant outstanding properties. In addition, CQDs have excellent electrochemical reaction performance due to their abundant quantity, low price, unique electron transfer capability, and large specific surface area. More importantly, CQDs can be doped with heteroatoms to change properties. For example, the fluorescence properties of CQDs can be changed by doping with heteroatoms. A facile and high-output strategy to fabricate selenium-doped carbon quantum dots (SeCQDs) [15] with green fluorescence was developed by the hydrothermal treatment of selenocystine under mild conditions. The selenium heteroatom imparts redoxdependent reversible fluorescence to Se-CQDs. Once Se-CQDs are internalized into cells, harmful high levels of reactive oxygen species (ROS) in the cells are reduced. With their fast electron transfer and large surface area, CQDs are also promising functional materials. Similarly, silicon and germanium nanostructures as high refractive index materials have been extensively studied as a new type of photoresonance structure. It is shown that silicon quantum dots (SQDs) can increase the internal potential of graphene/Si Schottky junctions and reduce the light reflection of photodetectors. Ting Yu et al. [16] achieved a faster response of photodetectors by coupling graphene with SQDs, and could further improve the performance of photodetectors by changing the size of silicon quantum dots and the number of graphene layers. Their excellent transmission and optical properties have potential applications in semiconductor lasers, amplifiers, and biosensors. Currently, the main method for the synthesis of quantum dots is the colloidal method. Colloidal synthesis involves heating the solution at a high temperature, decomposition of the precursor solution to form monomers, followed by nucleation, and formation of nanocrystals. This method can be used to synthesize quantum dots in large quantities. 

### 2.2. 1D Nanowires/Rods/Tubes

Nanowires are one-dimensional (1D) structural materials that are laterally confined to less than 100 nm. Compared to conventional bulk materials, nanowires tend to exhibit better photoelectric properties for macroscopic applications. For example, nanowires can naturally concentrate solar energy into a very small area of the crystal, concentrating light 15 times more intensely than ordinary light. This has important implications for the development of solar cells and the use of solar energy [17,18] because of the resonance of the light intensity within and around the nanowire crystal, which helps to increase the conversion efficiency of solar energy. Silver nanowire electrodes exhibit easily adjustable photoelectric and mechanical properties. Atomic-level chemical welding of silver nanowire electrodes [19] can be used to construct a flexible organic solar cell with high efficiency. Si/InP core-shell nanowire-based solar cell using etched Si nanowire [20] confirm the formation of radial nanowire heterostructures. In this cell, more photons can be absorbed. Compared to traditional solar cells, the performance is greatly improved.

CNTs are one of the highest hardness and best strength of synthetic carbon materials. In the CNTs, the C-C bonds are mainly sp^2^ hybridized, and the hexagonal mesh structure is bent to a certain extent, forming a spatial topology, in which certain sp^3^ hybridized bonds can be formed. sp^2^ hybridized C-C bonds are strong chemical bonds, which makes the CNTs have very high mechanical strength. For CNTs with an ideal monolith wall, its tensile strength is about 800 GPa. CNTs are also very flexible and can be stretched. The factor that usually determines the strength of a material is the aspect ratio, the ratio of length to diameter. If the aspect ratio reaches 20, it is an ideal flexible material. CNTs are flexible materials with high thermal conductivity because their aspect ratio can reach more than 1000 and their heat exchange performance along the length direction is very high. CNTs are also divided into single-walled carbon nanotubes (SWCNT) and multi-walled carbon nanotubes (MWCNT). The geometric structure of SWCNT can be regarded as a single layer of graphene crimp, with excellent electronic and mechanical properties. MWCNT is made of layers of graphene seamlessly coiled into concentric tubes. Compared with SWCNT, their elasticity and tensile strength are slightly inadequate. Nanofibers composed of a single polymer often have poor electrical conductivity and weak mechanical properties, so their applications are limited. Therefore, CNTs are often used as reinforcement fillers to prepare nanofibers after compounding with other polymers, which can effectively improve the properties of nanofibers. Fe_2_O_3_/C/CNT composites [21] sprayed by ultrasonic can be used as an anode for lithium-ion batteries. In these composites, the high conductivity of CNTs makes charge transfer faster, which improves the performance of lithium-ion batteries. The structure along the CNTs is the same as the sheet structure of graphite, so it also has very good electrical properties. Moreover, it has also realized excellent characteristics in thermal and optical aspects, so it has a very good prospect in battery, sensing, medical treatment, and other aspects. Similarly, silicon and germanium nanotubes are suitable to be used as anodes of lithium-ion batteries because of their cyclic stability. In recent years, further progress has been made on the crystal phase transformation of anode materials during battery charging and discharging, which is expected to improve the performance of lithium-ion batteries. It is worth mentioning that Chen et al. [22] deposited gold (Au), platinum (Pt), nickel (Ni), and indium tin oxide (ITO) onto the surface of tin dioxide nanotubes to prepare different kinds of electrodes. High sensitivity detection of hydrogen and benzene is achieved, and the power consumption is only 1% of that of commercial sensors.

### 2.3. Monolayer

Because of their excellent electronic, optical, and mechanical properties, two-dimensional (2D) nanomaterials graphene, silicene, and germanene have attracted wide interest. Graphene is a new material formed by a single layer of carbon atoms and is also the basic building block of other carbon-based nanomaterials, such as fullerenes, CNTs, and graphite. Graphene can be rolled into 0D fullerenes and 1D CNTs, or it can be stacked in a certain way to form a graphite bulk material. Graphene also exhibits remarkable physical properties due to its unique internal structure. 1. Graphene has excellent electronic properties [23]. It can carry a considerable amount of charged ions, so it is commonly used as a basic raw material for batteries and electrical equipment. 2. Graphene is so flexible that it can bend and fold to a certain extent with little change in its properties. Therefore, graphene has a very good prospect in the research field of flexible wearable electronic devices [24].

But the use of graphene is limited by its lack of a semiconductor band gap. Therefore, it is a difficult problem to study how to open the energy gap. At present, there are two main methods commonly used. The first one is to increase inherent defects of graphene, exposing more active sites. The quantum size effect of the electronic structure can be achieved by changing the morphology of graphene. For example, graphene can be changed into 0D graphene nanoribbons and 0D graphene quantum dots. The second is a chemical modification, which promotes the redistribution of charge on the surface by changing the number and type of heteroatoms incorporated into the graphene. The chemical modification includes surface modification and substitution doping. Graphene surface modification is achieved by hybrid adsorption of gaseous metals or organic molecules on the graphene surface. Alternating doping is the introduction of heteroatoms into the carbon lattice of graphene. At present, this modification method is very mature, and various elements have been widely introduced into graphene.

Monoatomic derivatives of graphene can be obtained by adding halogen atoms to the graphene skeleton. Graphene derivatives exhibit different properties due to the different electronegativity of heteroatoms. Among them, fluorographene has large negative magnetoresistance, high optical transparency, and high reactivity. As well, it is easy to generate many derivatives, such as graphene acid and cyanogen graphene. Graphene acid is a novel graphene platform whose carboxylic acid groups are selectively and uniformly located on the surface of the carbon network. This structure enables the graphene acid to have more uniform functionalization and stronger electron conduction. Such good performance also proves the excellent catalytic activity of graphene acid [25]. Furthermore, the selectivity of different oxidation products can be precisely modulated by adjusting the structure of the graphene acid. It is widely used in selective electrochemical sensing and catalysis [26]. Cyano graphene is also one of the graphene derivatives, capable of complex 2D chemical reactions and high yield covalent functionalization of graphene [27].

Since graphene was discovered in 2004, researchers have proposed silicene, germanene, and stanene with graphene-like honeycomb structures. Silicene has since been designed as a cathode to develop zinc-ion hybrid capacitors with enhanced capacitance and hypercyclic stability. As the research progressed, the researchers designed a hybrid honeycomb silicene, combining the electron band gap of specific silicon with the high electron mobility energy of honeycomb silicon. Zhao et al. confirmed that germanene is a potentially high energy density anode material. They prepared small layer germanene nanosheets by the liquid phase stripping method and measured their cyclic stability after mixing with rGO [28].

As mentioned above, we reviewed the different morphology and properties of IVA-LD. In fact, in research and application, they are not applied alone, but are often used in combination with other low-dimensional or bulk materials, which also shows the advantages of low-dimensional materials for easy composite. The composite of IVA-LD can not only have the excellent properties of each part of the material, but also forms heterojunction at the interface of the composite. Heterogeneous junctions form at the composite interface, among which the Van der Waals heterojunctions (vdWHs) formed by 2D materials stacking has attracted the researchers’ attention for the first time. Some studies show that vdWHs can provide the largest area for the separation and transfer of carriers, showing application potential in photoelectric detection. Some studies have shown that van der Waals heterojunction can provide the largest area for the separation and transfer of carriers, showing the potential of application in photoelectric detection. Dhungana et al. [29] introduced the concept of Xene heterostructures based on an epitaxial combination of silicene and stannene on Ag(111), promising to optimize the responsivity and speed of photodetectors.

## 3. Preparation of IVA-LD

### 3.1. Hydrothermal Synthesis

Hydrothermal synthesis is a simple synthesis method in which the prepared and stirred solution is put into an autoclave and reacted for a period at a certain temperature and pressure, using a hydrothermal system and a high-temperature and high-pressure closed environment to obtain IVA-LD. Many parameters, such as surfactant, solution pH value, reaction temperature, and reaction time need to be ensured in the experiment, which makes the product more sensitive to environmental changes. The advantages are high concentration, good dispersion, easy control of particle size, simple operation, large output, low cost, a mild and safe method of hydrothermal synthesis process, the sample reacts uniformly in an aqueous solution at a high rate under high pressure. Hydrothermal synthesis has shown great versatility and high efficiency in the preparation of holey materials.

**Figure 2:**
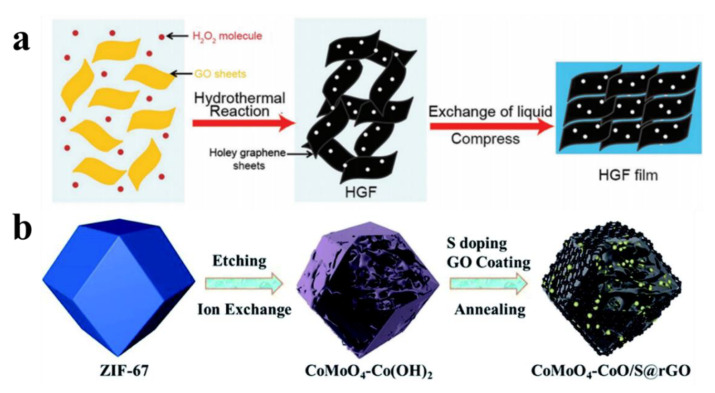
(**a**) Scheme of the synthetic process of HGFs and HGF films. Reprinted with permission from Ref. [29]. Copyright 2021 American Association for the Advanced Functional Materials. (**b**) Synthesis steps and structural description of CoMoO4–CoO/S@rGO NPs. Reprinted with permission from Ref. [31]. Copyright 2022 American Association for the Journal of Materials Chemistry A.

Xu et al. [30] synthesized porous graphene oxide (GO) frames with abundant planar nanopores through a solvothermal reaction involving a mild defect-etching process. A homogeneous aqueous mixture of GO and hydrogen peroxide was stirred and heated at 100 °C for 4 h to prepare a solution of hydrogen graphene oxide (HGO) (Figure 2a). The authors concluded that the oxidative etching reaction initiates and propagates mainly in oxygen-deficient regions, preferentially removing oxygen-containing carbon atoms, generating carbon vacancies, and eventually forming nanopores in the GO nanosheets. A simple metal-organic framework production strategy was proposed by Zhu et al. [31] Layered porous CoMoO_4_-CoO/S@rGO nanopolyhedra were synthesized by hydrothermal S-doping, as shown in Figure 2b. The preparation process can be divided into two parts; the first step synthesizes dodecahedral-shaped ZIF-67 crystal as the initial precursor. Secondly, the NPs of layered porous CoMoO_4_-Co(OH)_2_NPs were formed by injecting a dielectric sodium-molybdenum solution into the suspension of ZIF-67 crystals via the etching ion exchange effect. Then, TAA and GO solutions were added to the suspension of the above intermediates with CoMoO_4_-CoO/S@rGONPs, and the final products were obtained by low-temperature hydrothermal process and heat treatment hydrothermal process and heat treatment.

### 3.2. Template-Directed Synthesis

**Figure 3:**
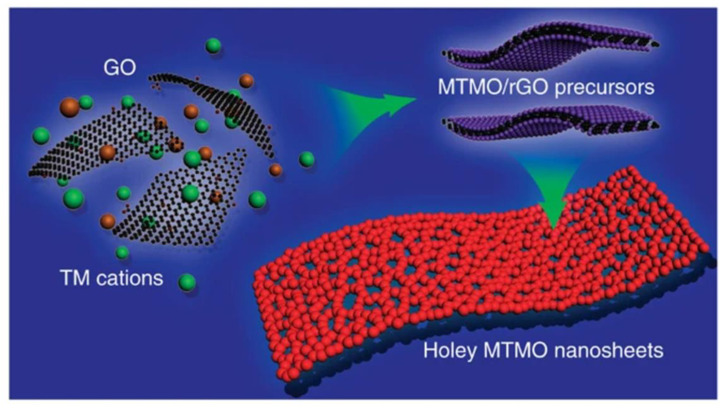
Schematic illustration of the general synthetic strategy of 2D holey TMO nanosheets. Reprinted with permission from Ref. [33]. Copyright 2012 American Association for the Advanced Energy Materials.

Template-directed synthesis is an efficient method for preparing multifunctional nanomaterials with multiple morphologies and structures because it allows direct tuning of the morphology and size of the nanomaterials by adjusting the preparation conditions and selecting a suitable template. A typical template growth process involves depositing or synthesizing a precursor on a substrate (template) and then removing the template through an etching process to produce porous nanosheet products [32]. For example, porous and polygonal magnesium oxide layers can be used as templates to obtain monolayer and bilayer porous graphene nanosheets. The resulting graphene nanosheets have a porous nanonet structure with a pore size distribution of 6–10 nm and an SSA of up to 1654 m^2^g^−1^ [33]. Sacrificial template guidance is an extension of the conventional template method and is a more effective way to prepare porous materials by applying a template as a precursor system. Graphene and its derivatives are important sacrificial templates for the synthesis of various ultra-thin, porous 2D nanosheets. Recently, Peng et al. proposed a general method for in-situ synthesis of 2D porous transition metal oxide (TMO) nanosheets [34]. Figure 3 shows that GO was first employed as a template to grow various TMO precursors on its surface, and then the TMO precursors are transformed into 2D porous TMO nanosheets after heat treatment due to the synergistic effect of chemical interconnection and GOs-controlled decomposition of TMO nanoparticles. Two transition metal (TM) cations are mixed with GO and then anchored on surfaces of the rGO template during solution-phase reaction. After removal of the rGO template during post-calcination, 2D porous MTMO nanosheets consisting of interconnected MTMO nanocrystals were formed.

### 3.3. Liquid Phase Stripping Nanosheet Method

**Figure 4:**
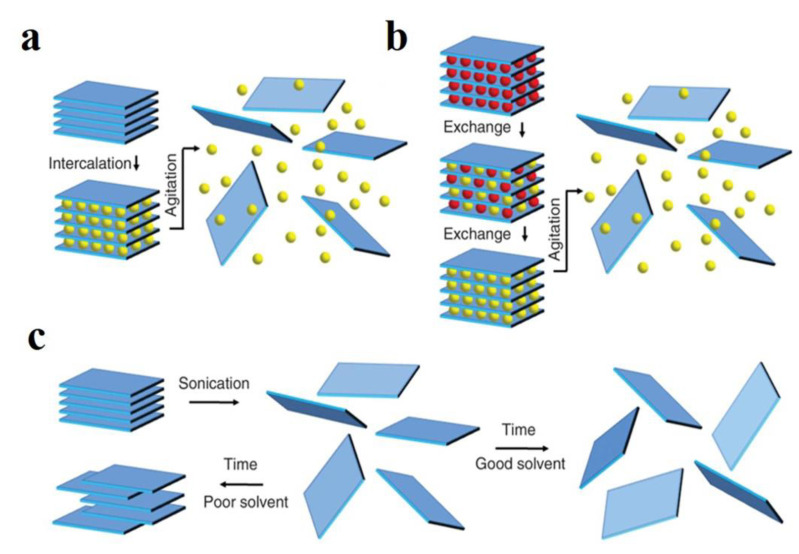
(**a**) Intercalation; (**b**) Ion exchange; (**c**) Sonication-aided exfoliation.

Based on the weak interlayer van der Waals interactions in layered compounds, a top-down synthesis method has been developed to overcome interlayer forces and to prepare 2D nanosheets by direct physical or chemical stripping from their bulk layered nanomaterials. High-quality micro-scale-width films have been obtained from bulk crystals using the sellotape method [35]. However, the process is time-consuming and difficult to control and is not suitable for the mass production of 2D nanosheets. To achieve high-quality large-scale synthesis of 2D nanosheets, as shown in Figure 4, liquid phase stripping of layered materials is usually carried out using the following three main methods: ion intercalation, ion exchange, and solvent ultrasonic treatment. Firstly, ion intercalation refers to the adsorption of guest molecules into the gap between layers, and this method is widely used in certain layered materials [36]. Ion intercalation usually increases the interval between layers, weakens the interlayer adhesion, and reduces energy, which is usually a disadvantage of ion intercalation methods. Also, another disadvantage of the ion intercalation method is that they are sensitive to environmental conditions [37]. However, ion intercalation methods are still under development and a large number of intercalation methods and intercalating agents are emerging. As scientific research continues, ion intercalation will play a greater role in the use of nanosheets.

Ion-exchange methods refer to the process of displacing ions between insoluble solid layered materials, such as Montmorillonite (MMT) and hydrotalcite, which normally carry exchangeable ions, and ions of the same charge in solution. In suspensions like MMT, for example, this layered structure combined with the unique and convenient migration of water molecules between layers allows ions to exchange with ions in body solution. The ion exchange between MMT and cations can peel off their layered structure, thus opening up a new avenue for novel 2D nanosheets [38]. The method lays the foundation for a general route to prepare large area monolayer nanosheets and the basic properties of 2D nanomaterials and develops a number of potential applications. 

The final presentation is an ultrasound-assisted liquid phase exfoliation strategy, which is also a popular method that is widely used due to its high yield. Ultrasonic generates cavitation bubbles or shear forces that separate layered material into monolayer to multilayer nanosheets. However, it also has many disadvantages such as poor structural integrity, size limitations, and low monolayer yield. This treatment destroys the layered microcrystalline structure and produces stripped nanosheets. The stability of ultrasound-treated nanosheets depends on various parameters and the choice of solvent is very important. As with MMT, it is difficult to separate monolayer nanosheets with 2D structures.

In addition, peeling materials with low reduction potentials, such as graphene, by adding hydroxyl and epoxy groups on its surface, produces hydrophilic properties that allow solvent water to be embedded and large-scale peeling, with dispersed sheets mainly in single layers, often spanning hundreds of nanometers stripping by extending the interlayer spacing. In addition to multi-step embedding, in-situ reactions of the embedding agent can be used to overcome interlayer forces and enable exfoliation. At a later stage, improved methods have been proposed to efficiently obtain high-quality graphene. In recent years, the controllability of the stripping process and the function of the product have been further developed based on the method of liquid phase stripping methods for the synthesis and practical application of 2D nanosheets of controllable quality. 

## 4. Applications of IVA-LD in Energy Conversion Materials

### 4.1. Battery

Classical graphene nanomaterials are good electronic conductors, with a zero-band gap structure and excellent electron transport capabilities making them good electrode materials. However, in many cases, graphene needs to be compounded with different materials to achieve fast electron and ion transport effects. Many examples have been reported of the design of hybrid structures of graphene with many oxides (Nb_2_O_5_, TiO_2_, MoO_3_, etc.) to achieve the mentioned functions [39,40,41]. Various carbon carriers such as nanotubes, graphene-based materials, and porous carbon not only act as electron channels, but also form heterojunctions between oxide and carbon atoms, thus influencing the electronic properties of both materials. Another way to achieve fast electron and ion transport is through the construction of 2D heterostructures, which facilitate the combination of highly conductive and high-energy density 2D materials. Since at least one material in the hybrid structure has good electrical conductivity, graphene is often the primary material of choice in this [42]. To date, this approach has been applied quite commonly and a large number of metallic conductors and active materials are also available. A class of 2D transition metal nitrides and carbides (MXenes) has been widely reported as a promising paradigm in the field of energy conversion and storage. Both MXene and graphene can be produced from their conventional materials using “top-down” stripping techniques (MAX material or graphite). This stripping method allows for the large-scale fabrication of ultra-thin 2D nanomaterials, down to a single atom or multiple atomic layers, resulting in a variety of unique chemical and physical properties. Second, ideal MXene and graphene materials have large specific surface areas and high electrical conductivity, making them excellent candidates for a variety of energy conversion and storage applications. In addition, heteroatoms (metallic or non-metallic) can be used to dope or modify the surface of the microstructure to improve performance. For example, a freestanding, ultra-lightweight, additive- and binder-free Ti_3_C_2_TxMXene was recently prepared by Olgani et al. [43]. It was shown that Ti_3_C_2_TxMXene aerogel could be aligned along a temperature gradient in the sub-millimeter region with a strain tolerance of up to 50%. MXene aerogel has an excellent electrochemical response, excellent rate performance, high specific capacity, and high cycle stability. This study shows that preventing re-stacking of MXene flakes during aerogel manufacturing eliminates the need for electrochemical cycling to achieve maximum volume. The excellent electromechanical properties of MXene aerogel result from the directional assembly of the 2D sheet in their structure, which makes them high-quality strain sensors. Zhang et al. [44] prepared a composite material containing MXene and SnS by a hydrothermal method. The introduction of SnS increases the interlayer spacing and enhances the reversibility and electrical conductivity of the composite. However, A pristine MoS_2_ electrode exhibits quick capacity attenuation and rate performance. A 2D composite material was prepared by Huang and coworkers [45] with the help of a hydrothermal technique. 

MoS_2_ nanosheets were introduced into the interlayer of Ti_3_C_2_T_x_MXene to create the composite. In a nutshell, the MXene@SnS and MXene@MoS_2_ composites exhibit outstanding electrochemical characteristics and have promising application possibilities due to the synergistic impact of SnS and MoS_2_ with high theoretical capacity and Ti_3_C_2_T_x_ with superior electrical conductivity.

Lithium-ion batteries (LIBs) dominate the power supply market for a wide range of devices, from electronics and new energy vehicles to networking applications [46]. The structure of IVA-LD plays an important role in improving the electrochemical performance of LIBs, such as power/energy density and cycling stability. Nanostructured electrodes that improve the overall performance of LIBs include ultra-thin, well-defined 2D nanomaterials, shortened lithium-ion transport channels, and abundant surface area for lithium-ion storage activity [47]. Despite the advantages of 2D nanomaterials in LIBs applications, the problem of self-filling of 2D nanomaterials in electrode manufacturing has been an impediment to their practical use in LIBs. During material processing or electrode fabrication, 2D nanomaterials can easily re-agglomerate into dense structures due to the weak van der Waals force between them [48], severely hindering electrolyte and ion penetration into the internal structure of the electrode and thus leading to rapid capacity decay.

**Figure 5:**
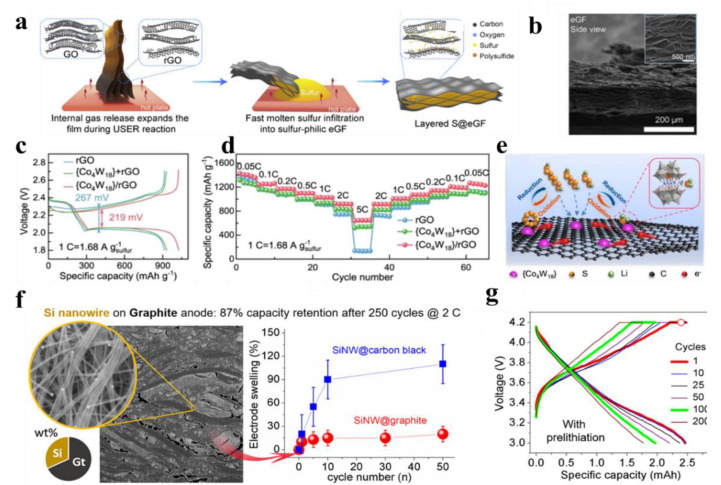
(**a**) Schematic diagrams; (**b**) SEM images for the expanded rGO film. Reprinted with permission from Ref. [48]. Copyright 2020 American Association for the Matter.; (**c**) Galvanostatic discharge–charge curves at 1 C (i.e., 1.68 A gs−1); (**d**) Rate performance of various sulfur cathodes at different specific current; (**e**) Schematic illustration of bifunctional catalytic effect for Li2S deposition and oxidation on the surface of Co4W18/rGO composites. Reprinted with permission from Ref. [49]. Copyright 2022 American Association for the Nature Communication; (**f**) Characterization of Gt−SiNW and Thickness and swelling percentage of electrodes vs. cycle number for SiNW@carbon black and SiNW@graphite; (**g**) prelithiation at C/2 rate. Comparison of corresponding reversible capacities, and Coulombic efficiency. Reprinted with permission from Ref. [75]. Copyright 2020 American Association for the ACS Nano.

Chen et al. have designed a horizontally aligned, high tortuosity porous rGO and used it as an efficient sulfur host [48]. Sulfur species can be firmly encapsulated in sandwiches of 2D carbon material, which can act as a barrier to suppress shuttle effects due to its inherent high conductivity and laminar flow confinement. These neatly aligned rGO nanosheets are made into sandwiches to limit the diffusion of dissolved LiPSs. The experimental results show that the curvature of rGO affects the inhibition of LiPSs on diffusion and dissolution. Higher electrode curvature may help ions to diffuse outward mass transfer paths to inhibit LiPSs diffusion from the cathode, as shown in Figure 5a,b. Based on these advantages, the cell achieved an ultra-high cathode area capacity of 21 mAh cm^−2^, after 160 cycles, with a capacity retention of 98.1%. Following this idea, the core concept of sulfur limitation in the conductive matrix can be further applied to the design of 3D frame hosts. rGO is a good carrier for coupling well with other materials, and Lei et al. [49] designed a single-dispersed molecular cluster catalyst composite comprising of a polyoxometalate framework [Co_4_(PW_9_O_34_)_2_]^10−^ shown in Figure 5e and a multilayer rGO. The composite demonstrates efficient polysulfides adsorption and reduced activation energy for polysulfides conversion due to interfacial charge transfer and exposure of unsaturated cobalt sites, making it highly advantageous for use as a bifunctional electrocatalyst. Figure 5c,d shows a significant increase in reactive polarization to 267 mV and 442 mV for the rGO/S cathode, and a slight increase in overpotential for the Co_4_W_18_/rGO/S cathode. Furthermore, the activation barrier of Li_2_S can be reduced on the Co_4_W_18_/rGO/S electrode compared to the rGO electrode, which further demonstrates the more significant oxidation kinetics of Li_2_S on the Co_4_W_18_/rGO/S cathode.

To improve the performance of the LIBs, the key is to develop novel electrode materials that could improve the energy density, extend the power capacity and prolong the life cycle [47,50]. Graphene/CNTs composites display great merits in the preparation of the anode materials for LIBs, and a lot of successes have been achieved by incorporating graphene/CNTs composites into LIBs. To improve the performance, Chen et al. [51] and Li et al. [52] synthesized Graphene/CNTs composites using CVD based methods and prepared lib based on these composites. These batteries are proven to have greater capacity, cyclability, and rate capability. The results show that the composite can achieve the highest thermal conductivity and temperature rise inhibition when the Graphene/CNT mass ratio is 7/3 [49], which indicates that the composite has great potential in the thermal management of lithium-ion power batteries. The bonding behavior between graphene and carbon nanotubes is of great significance to the electrical properties of composites. To further improve battery performance, different types of metals and metal oxide nanoparticles, Ni nanoparticles [53], Ge nanoparticles [54], V_2_O_5_ [37], SnO_2_ [55,56], Co_3_O_4_ [57], TiO_2_ [58] and MoS_2_ nanoparticles [59,60,61] are also added to the Graphene/CNTs composites to produce anode materials. These nanoparticle/Graphene/CNTs composites could be used to make anodes for LIBs with enhanced performance. Graphene/CNTs-based Si nanocomposites can also improve the performance of the active materials in LIBs. However, these candidates still produce severe capacity attenuation due to electrical disconnections and fractures caused by large volume changes in long cycles. Therefore, Tian et al. [62] designed a novel 3D crosslinked graphene and SWCNTs structure to encapsulate Si nanoparticles. The synthesized 3D structure is attributed to the excellent self-assembly of CNTs with GO and the heat treatment process at 900 °C. This special structure provides sufficient gap space for Si nanoparticles to expand in volume and provides channels for ion and electron diffusion. In addition, the cross-linking of graphene and SWCNTs also enhances the stability of the structure. As a result, the volume expansion of Si nanoparticles is limited. Specific capacity keeps at 1450 mAh g^−1^ after 100 cycles at 200 mA g^−1^. This well-defined 3D structure helps to achieve superior capacity and cycle stability compared to the mechanically mixed composite electrodes of bare silicon and graphene, single-walled carbon nanotubes, and silicon nanoparticles. In the same year, a porous Si/rGO/CNT composite was developed by facile chemical etching with a self-encapsulating process as anode material for full cell LIBs [63]. What is more, SnO_2_@carbon nanotube/reduced graphene oxide (SnO_2_@CNT/RGO) composite is rationally designed and fabricated [64], in which nano SnO_2_ nanoparticles (NPs, ~6 nm) are anchored onto 3D conductive CNTs/RGO skeleton by first assembling SnO_2_ onto CNTs and then entangling SnO_2_@CNT nanofibers in 3D graphene networks. The synergistic effect of CNTs and RGO significantly improved the conductivity and prevented the aggregation of active substances. In addition, the mesoporous structures constructed by CNTs and rGO can adapt to the volume changes of SnO_2_ NPs and form more stable SEI layers during repeated discharge/charge processes. Moreover, Graphene/CNTs composites could also promote the cathode function of LIBs [65,66,67,68] and inhibit the dendrite formation of the lithium anode of lithium-ion batteries [69], providing more opportunities for a huge increase in battery capacity and energy density. The addition of Graphene/CNTs composites helps to solve the existing problems such as slow reaction kinetics, polysulfide diffusion caused by insulating sulfur, and severe capacity loss, and further promotes the development of LIBs in the next generation of energy storage systems.

Silicon is also commonly used as a battery anode material, with a recording capacity (about 4000 mAhg^−1)^ more than ten times higher than the graphite used in commercial batteries [70]. As a result, silicon has attracted considerable interest in recent years as an anode material for lithium-ion batteries. However, the application of silicon is severely limited by its rapid degradation when used as an electrode, the potential for lithium/dehydrogenation processes to cause volume expansion effects of up to 300%, in addition to the numerous drawbacks faced by the electrode/electrolyte such as the instability of the interface and the low electrical conductivity of the material. To address these issues, scientists have explored many aspects. Most of these studies have attempted to come up with practical solutions using innovative electrode structures or silicon-based composites. They usually require silicon at the nanoscale (nanoparticles, core-shell structure, yolk-shell structure, nanoporous structure, nanowires, nanotubes, nanofibers, films, etc.) [71,72,73,74]. Nanostructured Si-based materials allow for high loading and cycling stability but remain a process and engineering. Haon et al. [75] designed a Si nanowires-grown-on-graphite one-pot composite (Gt−SiNW) via a simple and scalable route, as shown in Figure 5f. The uniform distribution of SiNW and the ordered arrangement of graphite flakes prevent electrode pulverization and accommodate volume expansion during cycling, resulting in very low electrode swelling. As shown in Figrue 5g, the Gt−SiNW anodes perform well in terms of ICE (72%), cyclability (900 mAhg^−1^ and 72% capacity retention at 300 cycles), rate capability (1145 mAhg^−1^ at 2 C rate), and extended cycling at high rate (629 mAhg^−1^ after 250 cycles at 2 C rate). It overcomes the technical hurdle of severe volume change with Si-rich anodes and exhibits an acceptable 20% electrode expansion after 50 cycles. This study found that graphite plays a key role in maintaining high energy density: it facilitates rapid electron transport and adaptation and directly influences the volume change during cycling, thereby improving long-term mechanical integrity.

Metallic tin-based materials have been a promising substitute due to their high specific capacity of up to 992 mAh g^−1^, proper lithium insertion potential, abundant natural resources, low price, and non-toxic and environmental friendliness. However, tin-based anodes suffer from an extreme volume expansion of up to 300% in the lithiation process (formation of Li_4.4_Sn). The severe volume change causes a significant structural collapse and an unstable SEI, resulting in substantial deterioration in the cycling performance [76]. However, similar to the silicon anode, the Sn anode suffers from a massive volume change due to a large amount of lithium insertion and extraction, which leads to pulverization of the electrode and loss of active material [77]. Li et al. [78] propose a shell-to-yolks evolution strategy to synthesize a novel structured Sn-based composite that can well address this issue. The as-prepared composite with multiple Sn cores embedded in one hollow nitrogen-doped carbon sphere is called multiple-yolks-shelled Sn@nitrogen doped carbon (MYS@Sn@NxC). The appropriate voids between Sn particles inside the sphere can well accommodate the volume change during cycles. As well, the robust NxC shell maintains a stable structure of the electrode. Moreover, through the metal-Sn synergistic effect, the volume expansion and rapid capacity decay for LIBs application can be effectively alleviated. Wang et al. [79] rationally design a Cu–Sn (e.g., Cu3Sn) intermetallic coating layer (ICL) to stabilize Sn through a structural reconstruction mechanism. The low activity of the Cu–Sn ICL against lithiation/delithiation enables the gradual separation of the metallic Cu phase from the Cu–Sn ICL, which provides a regulatable and appropriate distribution of Cu to buffer volume change of Sn anode. The proposed structural reconstruction mechanism is expected to open a new avenue for electrode stabilization for high-performance rechargeable batteries and beyond and more metal synergies need to be developed.

### 4.2. Transducer

The burning of fossil energy sources not only harms the environment, but also faces exhaustion. Therefore, the development of new, green non-polluting, and clean energy sources is a key to solving this problem. Water is ubiquitous in the atmosphere and has an amazing amount of energy. With the development of science and technology, obtaining clean energy from water has aroused great interest. We have found that water can interact directly with many functional materials to generate power. However, in the absence of chemisorption, the interaction between water vapor and solid surface is very weak. To enhance their interaction, it is necessary to change the composition of the surface structure or enlarge the effective area of the interaction. Nanomaterials are not only very small in size, but also have many tiny pore structures. It greatly increases the specific surface area of the nanomaterial considerably and promotes interaction with moisture. Moreover, nanomaterials are highly sensitive to external stimuli. It can therefore be optimized for functionalization by doping with other elements, changing the functional groups, or coupling to an external substrate. Nanomaterials, therefore, stand out among the materials used for the generation of electricity from water. Materials typically used for power generation mainly include carbon nanostructured materials.

**Figure 6:**
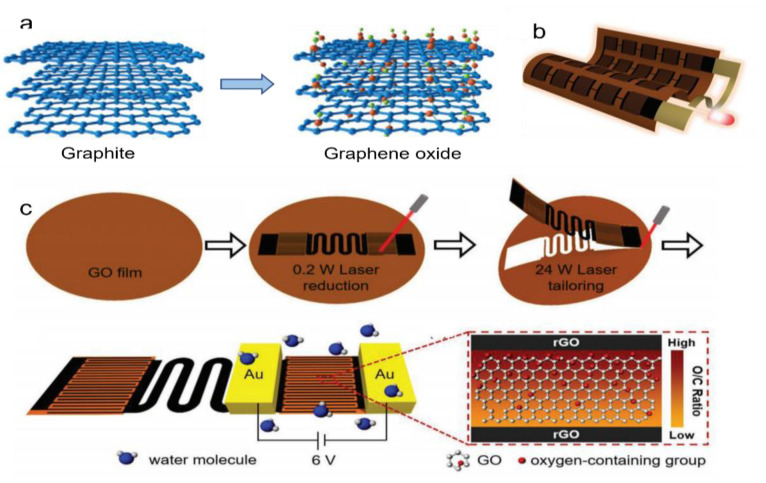
Electricity generation from graphene materials with instantaneous output. (**a**) Synthesis of GO from graphite through Hummers’ method. There are many oxygen functional groups on sheets of GO due to the strong oxidization of graphite. Reprinted with permission from Ref. [80]. Copyright 2018 American Association for Joural of Physics D-Applied Physics; (**b**) A large-scale, rollable HEG integration. (**c**) Schematic drawing of GHEG preparation. GO film and rGO interdigital electrodes and circuits that were in situ were reduced by direct laser writing. A pair of gold electrodes was physically pressed on rGO electrodes of GHEG and applied with a 6 V bias under a high-humidity environment. The oxygen-containing group distribution between the electrodes after the polarization process, which shows a concentration difference. Reprinted with permission from Ref. [82]. Copyright 2019 American Association for Advanced Materials.

The most prominent carbon nanomaterial in the field of moist-electric generation (MEG) is graphene, a monolayer of graphite with a honeycomb lattice. The chemical state of the graphene surface can be modified by oxygen-related functional groups [80], in which the graphite is oxidized and exfoliated to give GO, as shown in Figure 6a. In contrast to graphene, GO is modified by several oxygen-containing functional groups, such as -OH, -COOH. The addition of these functional groups increases the reactivity of graphene and greatly improves the hygroscopic and desorption properties when in contact with moisture [81]. It releases protons (H^+^) from the oxygen-containing functional groups, creating an ion gradient. The protons move from high to low concentrations, producing a stable voltage. There are therefore three main strategies to improve output performance.

**Figure 7:**
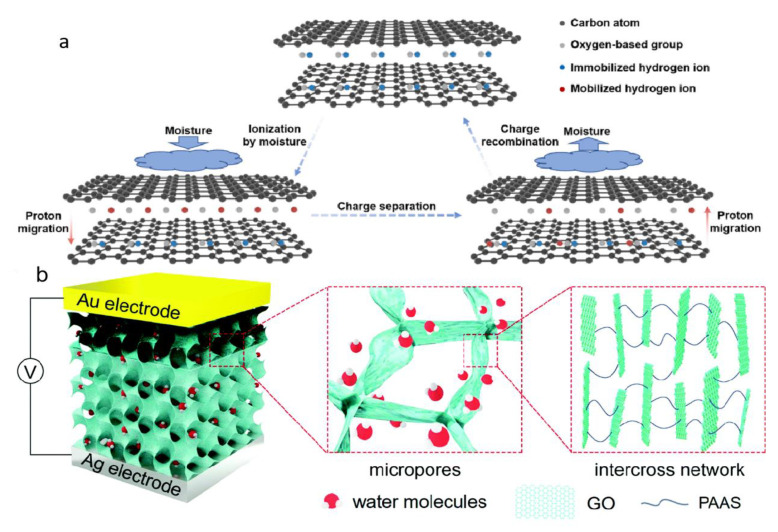
(**a**) Electric generation for acidified GO/PVA film. The protons in the functional groups of GO are mobilized by moisture absorption and achieve charge separation by proton migration toward the inner layer. Conversely, the migration direction is opposite under the moisture removal and contributes to the charge recombination. Reprinted with permission from Ref. [83]. Copyright 2022 American Association for Nano Energy; (**b**) Schematics of MEG. The MEG is composed of a pair of metal electrodes and porous GO composite with absorbed water molecules and heterogeneous chemical structure, where the darkened upside area was reduced by a directionally controlled laser. The GO composite has substantial micropores facilitating water molecule absorption and an abundant cross-linking network providing ion channels for fast carrier migration. Reprinted with permission from Ref. [84]. Copyright 2019 American Association for Energy&Environmental Science.

(1) Asymmetric treatment of material structure. As suggested by Yang et al., a potassium hydroxide (KOH) solution is added to the GO solution to introduce a large ionic gradient [81]. Most of the oxygen-containing functional groups of GO are destroyed by reaction with KOH. The structure of GO is destroyed, leaving potassium ions (K^+^) between the lamellar structures, forming rGO. GO and rGO come into contact through overlapping. An ionic solution is formed in the middle of the layered structure when exposed to moisture. So, the potassium ions (K^+^) are distributed asymmetrically throughout the system. The potassium ions (K^+^) move spontaneously from the rGO side to the GO side, generating a stable voltage and current. A graphene hygroelectric generator also has been prepared by laser treatment of graphene. The graphene hygroelectric generators can be folded [82], stretched, or even stripped in three dimensions. The rGO is formed by engraving the GO film using a direct laser writing technology, as in Figure 6b,c. On this basis, the gradient distribution of oxygen-containing groups between the positive and negative poles is changed by a moisture–electric annealing polarization process. When the device encounters moisture, the free hydrogen ions released from the oxygen-containing groups in it form an ion gradient and create a concentration difference within.
(2) Treatment of functional groups. GO can not only be reduced, but also acidified. After acidification, the density of functional groups on GO can be adjusted to make the functional groups dissociate more easily, resulting in a larger proton gradient difference between the upper and lower surfaces of the GO films. In zhu et al.’s. experiment [83], GO/PVA treated with 32% HCI could produce a voltage of 0.85 V. Apparently, acidification can greatly increase the voltage output, as shown in Figure 7a.

(3) Composite with other materials. GOs are also frequently combined with other materials to enhance their MEG function. Huang et al. proposed a moisture electric generator based on porous GO and PAAS composites [84], as shown in Figure 7b. In this material, the large specific surface area and hydrophilic groups work together to enhance its water absorption, which substantially promotes ion dissociation and efficient transport. In addition, the heterogeneous structure of the material and the asymmetric metal electrode allow the system to construct Schottky contact, which facilitates unidirectional ion transport and significantly improves the device performance. Carbon nanotubes can also be combined with GO. A MEG is fabricated by the end-to-end connection of two equal asymmetric regional sandwich structural GO/CNT composite films [85]. Proper addition of CNT helps to create continuous CNT network channels and generates a voltage as water flows over the CNTs surface, thus improving output performance. The generator uses exhaled moisture to generate electricity. As a person continues to breathe, electricity can be continuously generated.

### 4.3. Water Evaporation

In recent years, the photothermal conversion to obtain drinkable fresh water from abundant seawater has attracted great attention due to its energy source of inexhaustible solar energy, which is greener and more sustainable than other methods. Currently, the main factor limiting the application of photothermal conversion in drinking water is the low water evaporation rate, among which photothermal materials are the core devices, and the factors affecting the performance of photothermal materials are light absorption performance, photothermal conversion efficiency, thermal insulation, and water transport [86,87]. IVA-LD are mainly carbon and semiconductor materials. Carbon-based materials and semiconductor materials have received increasing attention from researchers as excellent photothermal materials. Carbon-based materials with π-π conjugate structure require a variety of doping in broadband absorption of sunlight compared with the semiconductor materials and carbon black materials have itself has an excellent absorption effect by themselves and are inexpensive and have great application advantages in this regard.

**Figure 8:**
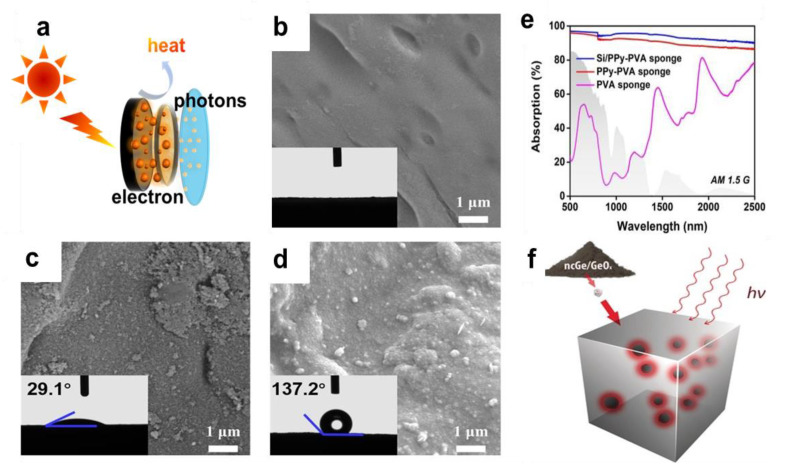
The fourth group of semiconductor nanomaterials is used as photothermal conversion materials. (**a**) Semiconductor photothermal conversion mechanism diagram; (**b**) PVA sponge fibers. Reprinted with permission from Ref. [89]. Copyright 2020 American Association for the Chemical Engineering Journal; (**c**) PPy-PVA sponge fibers.Reprinted with permission from Ref. [89]. Copyright 2020 American Association for the Chemical Engineering Journal; (**d**) Si/PPy-PVA sponge fibers. Reprinted with permission from Ref. [89]. Copyright 2020 American Association for the Chemical Engineering Journal; (**e**) UV–Vis–NIR absorption spectra of PVA sponge, PPy-PVA sponge, and Si/PPy-PVA sponge. Reprinted with permission from Ref. [89]. Copyright 2020 American Association for the Chemical Engineering Journal; (**f**) Photothermal transformation of germanium nanocrystals. Reprinted with permission from Ref. [92]. Copyright 2017 American Association for the Angewandte Chemie-International Edition.

Semiconductor materials absorb rapidly when exposed to sunlight, and the absorption of photons leads to the transition and relaxation of electrons (Figure 8a). During this process, there will be a great opportunity to release energy into the lattice and convert it into phonons, which will turn into heat for photothermal conversion. For example, monolithic tin monoselenide (SnSe), with its strong photo material coupling, wide absorption wavelength range, and outstanding quantum confinement effect, has great potential to effectively utilize solar radiation and convert it into heat [88]. Although silicon has been used for research in solar power generation due to its excellent photochemical properties, it is not suitable for use as a photothermal material for photothermal conversion due to its poor absorption of low-wavelength sunlight. Initially, the photothermal material was modified by loading silicon nanoparticles with silicon as a modifier [89], and the transformation of the material from hydrophilic to hydrophobic was achieved, as shown in Figure 8 b–d, which improved the absorption of sunlight (Figure 8e). Then by doping gold, silver, and other precious metals with silicon [90,91], the researchers realized the absorption of the full band of sunlight, which is used as a photothermal material generated by solar-thermal steam to study. Compared with silicon nanocrystals, germanium nanocrystals receive less attention, but have more excellent photothermal efficiency (Figrue 8f). Sun, et al. [92] prepared GeO by thermally induced dehydration of Ge(OH)_2_ to obtain size-controlled ncGe verified the superior photothermal performance of Ge nanocrystals over silicon nanocrystals, and expanded their research on its application in photothermal water evaporation and seawater desalination.

**Figure 9:**
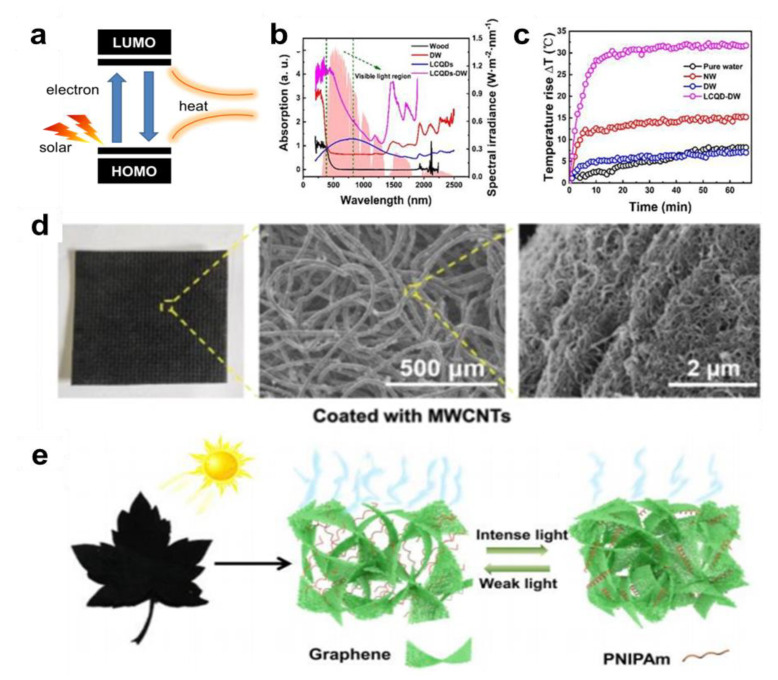
Carbon-based materials are used for photothermal conversion. (**a**) A schematic illustration of a carbon nanostructure with long internal optical-path lengths to enhance light absorption; (**b**) UV–Vis absorption of wood, DW, LCQDs, and LCQDs-DW. The standard AM 1.5 solar spectrum was set as the background and the visible light wavelength ranging from 380 nm to 780 nm was marked by a green dashed-line. Reprinted with permission from Ref. [95]. Copyright 2021 American Association for the Chemical Engineering Journal; (**c**) Temperature rising curve for pure water, wood, DW, and LCQDs-DW. Reprinted with permission from Ref. [95]. Copyright 2021 American Association for the Chemical Engineering Journal; (**d**) Photographs and SEM images of nonwoven (inset is the cross-section SEM image of PP/PE fiber) with MWCNTs. Reprinted with permission from Ref. [98]. Copyright 2021 American Association for the Advanced Science; (**e**) Under the different intensity of solar irradiation, the water transport channels can be autonomously turned on and off by the opening and closing of microstructures. Reprinted with permission from Ref. [100]. Copyright 2018 American Association for the Angewandte Chemie-International Edition.

The natural black color of carbon-based materials allows them to absorb sunlight at a wide wavelength range. The π-π conjugated structure is widely present in carbon-based materials, allowing excited electrons to jump from the highest occupied molecular orbit (HOMO) to the lowest unoccupied molecular orbit (LUMO) after absorbing photons and then return to the ground state orbit by releasing heat, as shown in Figrue 9a. Compared with homologous semiconductor materials, carbon-based materials have significant advantages such as excellent light absorption and photothermal conversion properties, easy processing, and low price. They have a wide range of promising applications in photothermal conversion, evaporation, and desalination of water. In recent years, researchers have conducted extensive and in-depth research on carbon-based materials of different dimensions, such as carbon quantum dots, carbon nanotubes, graphene nanosheets, etc. As an excellent dopant, CQDs were firstly doped into the permeable membrane [93,94], which showed excellent deconfliction and purification ability and increased pure water flux, drawing the attention of researchers. After that, researchers introduced CQDs into photothermal devices, which can improve the photothermal conversion performance of photothermal materials while solving the problem of desalination and decontamination. Chao et al. [95] loaded carbon quantum dots (LCQD) prepared by hydrothermal method onto delignified wood (DW) substrates to improve the photothermal materials, and their sunlight absorption performance and photothermal performance were improved, as shown in Figure 9b,c. The longitudinal and transverse anisotropy of thermal conductivity of macroscopic carbon nanotube arrangement [96] makes it a natural photothermal material with excellent thermal management by integrating external and main water insulation and internal water evaporation heat conduction. Chen et al. [97] pioneered the coating of CNTs on chemically treated wood substrates to realize a photothermal device that integrates sunlight absorption, heat management, and water transmission. Using the unique unidirectional water permeability property of the all-fiber structure, Zhu et al. [98] loaded CNTs onto the substrate to achieve water transmission control, thus promoting the increase of water evaporation rate, as shown in Figure 9d. Later, Zhao et al. [99] integrated CNTs with SMP to achieve flexible folding of photothermal devices and achieved thermal management of sunlight absorption due to the presence of carbon nanotubes. IVA-LD have significant advantages in material integration to improve performance. Graphene is an excellent photothermal material due to its excellent mechanical properties such as high toughness, better thermal conductivity than CNTs and excellent optical properties, and easy modification and assembly. By introducing thermally responsive PNIPAm into the microporous graphene frames, the biomimetic materials prepared by Zhang P et al. [100], achieved reversible regulation of pore size and hydrophilicity under different lighting conditions, and realized self-regulation of water for evaporation, as shown in Figure 9e. Cui et al. combined graphene photothermal materials with solar cells to achieve the combined photoelectric thermal effect, and the water evaporation rate reached the highest rate reported at that time 2.01–2.61 kg m^−2^ h^−1^ at 1 sun. More importantly, the integrated utilization of the photoelectric thermal effect broadened the application potential of graphene as a photothermal material. In particular, Lu et al. [101] used graphene materials for membrane distillation and successfully developed a catalytic pyrolysis process for the preparation of ultrathin NG membrane from solid carbon sources. The prepared graphene with high porosity atomic layer thickness, combined with the hydrophobic property of graphene naturally implements the vapor high selectivity, high flux through the graphene membrane, and the salt intercept (>99.8%). Meanwhile, the photothermal properties of graphene were utilized to achieve a temperature difference of 65/25 °C on both sides of the membrane under solar irradiation to achieve a high flux of LMH for water purification, which is much higher than the membrane flux (<80 LMH) currently reported in natural mode.

## 5. Summary and Prospects

In this review, we systematically introduce IVA-LD, mainly carbon-based nanomaterials and semiconductor nanomaterials, and their different morphologies and unique properties. The last few years have witnessed a wide range of applications of IVA-LD in energy conversion due to their unique mechanical, optical, electrochemical, and thermal properties. In addition, recent advances in the synthesis and preparation methods of IVA-LD are reviewed. Also, new synthesis and preparation methods have promoted the application of IVA-LD in energy conversion to a certain extent. We present in detail the progress of applications in battery energy storage, MEG, and photothermal evaporation.

Group IVA elements with similar valence electron structures exhibit efficient solar-thermal conversion properties, especially carbon-based materials that have been widely used in battery, MEG, and photothermal evaporation in recent years due to their diverse morphology and low price and are expected to be commercialized in a green way. In the aspect of battery, the application of carbon materials and silicon materials in electrode materials is explored. The doping of some transition metals will significantly improve their performance, and starting with cathode materials, inhibiting the dissolution of active substances in the direction of future research. In the aspect of MEG, the voltage and current are caused by the directional motion of charged particles driven by concentration gradient. Some methods to improve the power generation performance, such as increasing specific surface area and modifying their surfaces, are also discussed. As the understanding and research of the material have improved, the voltage output has been increasing while becoming more stable. MEG have been widely used in sensors and self-powered electronic devices. For efficient photothermal evaporation, it is important to realize the broad-band solar energy absorption, heat insulation management, and water transport management simultaneously in photothermal materials. IVA-LD are easy to process and load, and easy to integrate with other materials to add, photothermal management, while retaining the properties of other materials, thus enabling broad-band solar energy absorption, heat insulation management, and water transport management as one of the photothermal materials. These characteristics enable biomimetic materials, foldable flexible materials and devices integrated with solar cells, with potential applications in solar-thermal evaporation. Although good results have been achieved in battery, MEG, and photothermal evaporation, many aspects are still not perfect, and many problems need to be solved.

Here is a summary of what we can work on and the challenges ahead. Increasing the cycle stability and safety, power output, and water yield remains a top priority. While the cycle stability and safety, voltage output, and water yield has been improved, it is nowhere near enough to scale production. It still does not meet our most pressing needs. On the one hand, we consider exploring the properties of IVA-LD, where the combination of pore structures of 3D porous materials as intelligent and integrated materials will be their future direction. On the other hand, it is also important to find new materials that can be used for energy conversion. There is a wide variety of materials in nature, and many potential resources remain untapped. The discovery of new materials may lead to new directions for battery energy storage, MEG, and photothermal evaporation.

## Data Availability

Not applicable.

## References

[1] Kittner N., Lill F., Kammen D.M. (2017). Energy storage deployment and innovation for the clean energy transition. Nat. Energy.

[2] Arent D.J., Bragg-Sitton S.M., Miller D.C., Tarka T.J., Garfield D.J. (2020). Multi-input, Multi-output Hybrid Energy Systems. Joule.

[3] Chu S., Cui Y., Liu N. (2017). The path towards sustainable energy. Nat. Mater..

[4] Ajayan P.M. (2019). The nano-revolution spawned by carbon. Nature.

[5] Marrows C.H. (2021). Silicon goes heavyweight. Nat. Mater..

[6] Fadaly E.M.T., Dijkstra A., Suckert J.R., Ziss D., van Tilburg M.A.J., Mao C., Ren Y., van Lange V.T., Korzun K., Kölling S. (2020). Direct-bandgap emission from hexagonal Ge and SiGe alloys. Nature.

[7] Farquhar A.K., Supur M., Smith S.R., Dyck C.V., McCreery R.L. (2018). Hybrid Graphene Ribbon/Carbon Electrodes for High-Performance Energy Storage. Adv. Energy Mater..

[8] Zhang X.S., Kwon K., Henriksson J., Luo J., Wu M.C. (2022). A large-scale microelectromechanical-systems-based silicon photonics LiDAR. Nature.

[9] Luo W., Gong Y.H., Zhu Y.Z., Li Y.J., Yao Y.G., Zhang Y., Fu K., Pastel G., Lin C.F., Mo Y.J. (2017). Reducing Interfacial Resistance between Garnet-Structured Solid-State Electrolyte and Li-Metal Anode by a Germanium Layer. Adv. Mater..

[10] Kang L.T., Cui M.W., Jiang F.Y., Gao Y.F., Luo H.J., Liu J.J., Liang W., Zhi C.Y. (2018). Nanoporous CaCO_3_ Coatings Enabled Uniform Zn Stripping/Plating for Long-Life Zinc Rechargeable Aqueous Batteries. Adv. Energy Mater..

[11] Li Y.Y., Chen N., Li Z.L., Shao H.B., Sun X.T., Liu F., Liu X.T., Guo Q., Qu L.T. (2021). Reborn Three-Dimensional Graphene with Ultrahigh Volumetric Desalination Capacity. Adv. Mater..

[12] Stetson C., Schnabel M., Li Z.F., Harvey S.P., Jiang C.S., Norman A., Decaluwe S.C., Al-Jassim M., Burrell A. (2020). Microscopic Observation of Solid Electrolyte Interphase Bilayer Inversion on Silicon Oxide. ACS Energy Lett..

[13] Zhang C.Y., Liang S.X., Liu W., Eickemeyer F.T., Cai X.B., Zhou K., Bian J., Zhu H.W., Zhu C., Wang N. (2021). Ti1-graphene single-atom material for improved energy level alignment in perovskite solar cells. Nat. Energy.

[14] Pomerantseva E., Bonaccorso F., Feng X., Cui Y., Gogotsi Y. (2019). Energy storage: The future enabled by nanomaterials. Science.

[15] Li F., Li T., Sun C., Xia J., Jiao Y., Xu H. (2017). Selenium-Doped Carbon Quantum Dots for Free-Radical Scavenging. Angew. Chem. Int. Ed..

[16] Yu T., Wang F., Xu Y., Ma L., Pi X., Yang D. (2016). Graphene Coupled with Silicon Quantum Dots for High-Performance Bulk-Silicon-Based Schottky-Junction Photodetectors. Adv. Mater..

[17] Zhang B., Jie J., Zhang X., Ou X., Zhang X. (2017). Large-Scale Fabrication of Silicon Nanowires for Solar Energy Applications. ACS Appl. Mater. Interf..

[18] Peng K.Q., Lee S.T. (2011). Silicon Nanowires for Photovoltaic Solar Energy Conversion. Adv. Mater..

[19] Zeng G., Chen W., Chen X., Hu Y., Chen Y., Zhang B., Chen H., Sun W., Shen Y., Li Y. (2022). Realizing 17.5% Efficiency Flexible Organic Solar Cells via Atomic-Level Chemical Welding of Silver Nanowire Electrodes. J. Am. Chem. Soc..

[20] Pal B., Sarkar K.J., Banerji P. (2020). Fabrication and studies on Si/InP core-shell nanowire based solar cell using etched Si nanowire arrays. Sol. Energy Mater Sol. Cells.

[21] Park C., Samuel E., Joshi B., Kim T., Aldalbahi A., El-Newehy M., Yoon W.Y., Yoon S.S. (2020). Supersonically sprayed Fe2O3/C/CNT composites for highly stable Li-ion battery anodes. Chem. Eng. J..

[22] Chen J., Chen Z., Boussaid F., Zhang D., Pan X., Zhao H., Bermak A., Tsui C.Y., Wang X., Fan Z. (2018). Ultra-Low-Power Smart Electronic Nose System Based on Three-Dimensional Tin Oxide Nanotube Arrays. ACS Nano.

[23] Ye M., Zhang Z., Zhao Y., Qu L. (2018). Graphene Platforms for Smart Energy Generation and Storage. Joule.

[24] Liang Y., Zhao F., Cheng Z.H., Zhou Q.H., Shao H.B., Jiang L., Qu L.T. (2017). Self-powered wearable graphene fiber for information expression. Nano Energy.

[25] Blanco M., Mosconi D., Otyepka M., Medved M., Bakandritsos A., Agnoli S., Granozzi G. (2019). Combined high degree of carboxylation and electronic conduction in graphene acid sets new limits for metal free catalysis in alcohol oxidation. Chem Sci.

[26] Chronopoulos D.D., Bakandritsos A., Pykal M., Zboril R., Otyepka M. (2017). Chemistry, properties, and applications of fluorographene. Appl Mater Today.

[27] Bakandritsos A., Pykal M., Blonski P., Jakubec P., Chronopoulos D.D., Polakova K., Georgakilas V., Cepe K., Tomanec O., Ranc V. (2017). Cyanographene and Graphene Acid: Emerging Derivatives Enabling High-Yield and Selective Functionalization of Graphene. ACS Nano.

[28] Zhao F., Wang Y., Zhang X., Liang X., Zhang F., Wang L., Li Y., Feng Y., Feng W. (2020). Few-layer methyl-terminated germanene–graphene nanocomposite with high capacity for stable lithium storage. Carbon.

[29] Dhungana D.S., Grazianetti C., Martella C., Achilli S., Fratesi G., Molle A. (2021). Two-Dimensional Silicene–Stanene Heterostructures by Epitaxy. Adv. Funct. Mater..

[30] Xu Y., Chen C.Y., Zhao Z., Lin Z., Lee C., Xu X., Wang C., Huang Y., Shakir M.I., Duan X. (2015). Solution Processable Holey Graphene Oxide and Its Derived Macrostructures for High-Performance Supercapacitors. Nano Lett.

[31] Chen J., Zhu K., Liang P., Wu M., Rao Y., Zheng H., Liu J., Yan K., Wang J. (2022). Ultrahigh reversible lithium storage of hierarchical porous Co–Mo oxides via graphene encapsulation and hydrothermal S-doping. J. Mater. Chem. A.

[32] Bols P.S., Anderson H.L. (2018). Template-Directed Synthesis of Molecular Nanorings and Cages. Acc. Chem. Res..

[33] Fan Z., Liu Y., Yan J., Ning G., Wang Q., Wei T., Zhi L., Wei F. (2012). Template-Directed Synthesis of Pillared-Porous Carbon Nanosheet Architectures: High-Performance Electrode Materials for Supercapacitors. Adv. Energy Mater..

[34] Peng L., Xiong P., Ma L., Yuan Y., Zhu Y., Chen D., Luo X., Lu J., Amine K., Yu G. (2017). Holey two-dimensional transition metal oxide nanosheets for efficient energy storage. Nat. Commun..

[35] Geim A.K. (2009). Graphene: Status and Prospects. Science.

[36] Dreyer D.R., Park S., Bielawski C.W., Ruoff R.S. (2010). The chemistry of graphene oxide. Chem. Soc. Rev..

[37] Backes C., Higgins T.M., Kelly A., Boland C., Harvey A., Hanlon D., Coleman J.N. (2017). Guidelines for Exfoliation, Characterization and Processing of Layered Materials Produced by Liquid Exfoliation. Chem. Mater..

[38] Walker G.F., Garrett W.G. (1967). Chemical Exfoliation of Vermiculite and the Production of Colloidal Dispersions. Science.

[39] Wang X., Li Q., Zhang L., Hu Z., Yu L., Jiang T., Lu C., Yan C., Sun J., Liu Z. (2018). Caging Nb_2_O_5_ Nanowires in PECVD-Derived Graphene Capsules toward Bendable Sodium-Ion Hybrid Supercapacitors. Adv. Mater..

[40] Gao L., Cao M., Fu Y.Q., Zhong Z., Shen Y., Wang M. (2016). Hierarchical TiO_2_ spheres assisted with graphene for a high performance lithium–sulfur battery. J. Mater. Chem. A.

[41] Wang S., Zhang H., Zhang D., Ma Y., Bi X., Yang S. (2018). Vertically oriented growth of MoO_3_ nanosheets on graphene for superior lithium storage. J. Mater. Chem. A.

[42] Ma C., Liao Q., Sun H., Lei S., Zheng Y., Yin R., Zhao A., Li Q., Wang B. (2018). Tuning the Doping Types in Graphene Sheets by N Monoelement. Nano Lett..

[43] Orangi J., Tetik H., Parandoush P., Kayali E., Lin D., Beidaghi M. (2021). Conductive and highly compressible MXene aerogels with ordered microstructures as high-capacity electrodes for Li-ion capacitors. Mater. Today Adv..

[44] Zhang Y., Guo B., Hu L., Xu Q., Li Y., Liu D., Xu M. (2018). Synthesis of SnS nanoparticle-modified MXene (Ti_3_C_2_Tx) composites for enhanced sodium storage. J. Alloys Compd..

[45] Huang H., Cui J., Liu G., Bi R., Zhang L. (2019). Carbon-Coated MoSe_2_/MXene Hybrid Nanosheets for Superior Potassium Storage. ACS Nano.

[46] Manthiram A. (2017). An Outlook on Lithium Ion Battery Technology. An Outlook on Lithium Ion Battery Technology. ACS Cent. Sci..

[47] Li M., Lu J., Chen Z., Amine K. (2018). 30 Years of Lithium-Ion Batteries. Adv. Mater..

[48] Chen H., Zhou G., Boyle D., Wan J., Wang H., Lin D., Mackanic D., Zhang Z., Kim S.C., Lee H.R. (2020). Electrode Design with Integration of High Tortuosity and Sulfur-Philicity for High-Performance Lithium-Sulfur Battery. Matter.

[49] Lei J., Fan X.X., Liu T., Xu P., Hou Q., Li K., Yuan R.M., Zheng M.S., Dong Q.F., Chen J.J. (2022). Single-dispersed polyoxometalate clusters embedded on multilayer graphene as a bifunctional electrocatalyst for efficient Li-S batteries. Nat. Commun..

[50] Nitta N., Wu F., Lee J.T., Yushin G. (2015). Li-ion battery materials: Present and future. Mater. Today.

[51] Chen S., Chen P., Wang Y. (2011). Carbon nanotubes grown in situ on graphene nanosheets as superior anodes for Li-ion batteries. Nanoscale.

[52] Li S., Luo Y., Lv W., Yu W., Wu S., Hou P., Yang Q., Meng Q., Liu C., Cheng H.-M. (2011). Vertically Aligned Carbon Nanotubes Grown on Graphene Paper as Electrodes in Lithium-Ion Batteries and Dye-Sensitized Solar Cells. Adv. Energy Mater..

[53] Bae S.-H., Karthikeyan K., Lee Y.-S., Oh I.-K. (2013). Microwave self-assembly of 3D graphene-carbon nanotube-nickel nanostructure for high capacity anode material in lithium ion battery. Carbon.

[54] Fang S., Shen L., Zheng H., Zhang X. (2015). Ge–graphene–carbon nanotube composite anode for high performance lithium-ion batteries. J. Mater. Chem. A.

[55] Ma C., Jiang J., Han Y., Gong X., Yang Y., Yang G. (2019). The composite of carbon nanotube connecting SnO_2_/reduced graphene clusters as highly reversible anode material for lithium-/sodium-ion batteries and full cell. Compos. B. Eng..

[56] Woo H., Wi S., Kim J., Kim J., Lee S., Hwang T., Kang J., Kim J., Park K., Gil B. (2018). Complementary surface modification by disordered carbon and reduced graphene oxide on SnO_2_ hollow spheres as an anode for Li-ion battery. Carbon.

[57] Asiri A.M., Akhtar K., Khan S.B. (2019). Cobalt oxide nanocomposites and their electrocatalytic behavior for oxygen evolution reaction. Ceram. Int..

[58] Luo J., Li F., Zhou Y., Liu S., Ma J., Liu J. (2018). Paper-like TiO_2_/graphene-carbon nanotube hybrid electrode with high mass loading: Toward high-performance lithium ion battery. J. Alloys Compd..

[59] Ren J., Ren R.-P., Lv Y.-K. (2018). A flexible 3D graphene@CNT@MoS_2_ hybrid foam anode for high-performance lithium-ion battery. Chem. Eng. J..

[60] Zhai X., Mao Z., Zhao G., Rooney D., Zhang N., Sun K. (2018). Nanoflake δ-MnO_2_ deposited on carbon nanotubes-graphene-Ni foam scaffolds as self-standing three-dimensional porous anodes for high-rate-performance lithium-ion batteries. J. Power Sources.

[61] Ma H., Du S., Tao H., Li T., Zhang Y. (2018). Three-dimensionally integrated carbon tubes/MoS_2_ with reduced graphene oxide foam as a binder-free anode for lithium ion battery. J. Electroanal. Chem..

[62] Tian S., Zhu G., Tang Y., Xie X., Wang Q., Ma Y., Ding G., Xie X. (2018). Three-dimensional cross-linking composite of graphene, carbon nanotubes and Si nanoparticles for lithium ion battery anode. Nanotechnology.

[63] Gao X., Wang F., Gollon S., Yuan C. (2018). Micro Silicon–Graphene–Carbon Nanotube Anode for Full Cell Lithium-ion Battery. J. Electrochem. Energy Convers. Storage.

[64] Wang M.-S., Wang Z.-Q., Jia R., Yang Z.-L., Yang Y., Zhu F.-Y., Huang Y., Li X. (2018). Nano tin dioxide anchored onto carbon nanotube/graphene skeleton as anode material with superior lithium-ion storage capability. J. Electroanal. Chem..

[65] Cheng D., Zhao Y., Tang X., An T., Wang X., Zhou H., Zhang D., Fan T. (2019). Densely integrated Co, N-Codoped Graphene@Carbon nanotube porous hybrids for high-performance lithium-sulfur batteries. Carbon.

[66] Shi H., Zhao X., Wu Z.-S., Dong Y., Lu P., Chen J., Ren W., Cheng H.-M., Bao X. (2019). Free-standing integrated cathode derived from 3D graphene/carbon nanotube aerogels serving as binder-free sulfur host and interlayer for ultrahigh volumetric-energy-density lithiumsulfur batteries. Nano Energy.

[67] Wang N., Peng S., Chen X., Wang J., Wang C., Qi X., Dai S., Yan S. (2019). Construction of ultrathin MnO_2_ decorated graphene/carbon nanotube nanocomposites as efficient sulfur hosts for high-performance lithium–sulfur batteries. RSC Adv..

[68] Geng X., Yi R., Yu Z., Zhao C., Li Y., Wei Q., Liu C., Zhao Y., Lu B., Yang L. (2018). Isothermal sulfur condensation into carbon nanotube/nitrogen-doped graphene composite for high performance lithium–sulfur batteries. J. Mater. Sci. Mater. Electron..

[69] Raji A.-R.O., Villegas Salvatierra R., Kim N.D., Fan X., Li Y., Silva G.A.L., Sha J., Tour J.M. (2017). Lithium Batteries with Nearly Maximum Metal Storage. ACS Nano.

[70] Ozanam F., Rosso M. (2016). Silicon as anode material for Li-ion batteries. Mater. Sci. Eng. B.

[71] Gu M., He Y., Zheng J., Wang C. (2015). Nanoscale silicon as anode for Li-ion batteries: The fundamentals, promises, and challenges. Nano Energy.

[72] Du F.-H., Wang K.-X., Chen J.-S. (2016). Strategies to succeed in improving the lithium-ion storage properties of silicon nanomaterials. J. Mater. Chem. A.

[73] Ko M., Chae S., Cho J.J.C. (2015). Challenges in accommodating volume change of Si anodes for Li-ion batteries. ChemElectroChem.

[74] Grigoras K., Keskinen J., Grönberg L., Yli-Rantala E., Laakso S., Välimäki H., Kauranen P., Ahopelto J., Prunnila M. (2016). Conformal titanium nitride in a porous silicon matrix: A nanomaterial for in-chip supercapacitors. Nano Energy.

[75] Karuppiah S., Keller C., Kumar P., Jouneau P.H., Aldakov D., Ducros J.B., Lapertot G., Chenevier P., Haon C. (2020). A Scalable Silicon Nanowires-Grown-On-Graphite Composite for High-Energy Lithium Batteries. ACS Nano.

[76] Jing W.T., Yang C.C., Jiang Q. (2020). Recent progress on metallic Sn- and Sb-based anodes for sodium-ion batteries. J. Mater. Chem. A.

[77] Park M.-G., Lee D.-H., Jung H., Choi J.-H., Park C.-M. (2018). Sn-Based Nanocomposite for Li-Ion Battery Anode with High Energy Density, Rate Capability, and Reversibility. ACS Nano.

[78] Li B., Zhang T., Wei S., Gao W. (2022). Nitrogen-doped carbon hollow spheres packed with multiple nano Sn particles for enhanced lithium storage. Chem. Eng. J..

[79] Wang G., Aubin M., Mehta A., Tian H., Chang J., Kushima A., Sohn Y., Yang Y. (2020). Stabilization of Sn Anode through Structural Reconstruction of a Cu–Sn Intermetallic Coating Layer. Adv. Mater..

[80] Chen D., Tang J., Zhang X., Fang J., Li Y., Zhuo R. (2018). Detecting decompositions of sulfur hexafluoride using reduced graphene oxide decorated with Pt nanoparticles. J. Phys. D.

[81] Yang L., Yang F., Liu X., Li K., Zhou Y., Wang Y., Yu T., Zhong M., Xu X., Zhang L. (2021). A moisture-enabled fully printable power source inspired by electric eels. Proc. Natl. Acad. Sci. USA.

[82] Yang C., Huang Y., Cheng H., Jiang L., Qu L. (2019). Hygroelectric Generators: Rollable, Stretchable, and Reconfigurable Graphene Hygroelectric Generators. Adv. Mater..

[83] Zhu R., Zhu Y., Chen F., Patterson R., Zhou Y., Wan T., Hu L., Wu T., Joshi R., Li M. (2022). Boosting moisture induced electricity generation from graphene oxide through engineering oxygen-based functional groups. Nano Energy.

[84] Xu T., Ding X., Huang Y., Shao C., Song L., Gao X., Zhang Z., Qu L. (2019). An efficient polymer moist-electric generator. Energy Environ. Sci..

[85] Chen S., Xia H., Ni Q.-Q. (2022). A sustainable, continuously expandable, wearable breath moisture-induced electricity generator. Carbon.

[86] Lu Q., Shi W., Yang H., Wang X.J.A.M. (2020). Nanoconfined Water-Molecule Channels for High-Yield Solar Vapor Generation under Weaker Sunlight. Adv. Mater..

[87] Liang H., Liao Q., Chen N., Liang Y., Lv G., Zhang P., Lu B., Qu L.J.A.C. (2019). Thermal Efficiency of Solar Steam Generation Approaching 100% through Capillary Water Transport. Angew. Chem. Int. Ed..

[88] Yao J., Zheng Z., Yang G. (2018). Layered tin monoselenide as advanced photothermal conversion materials for efficient solar energy-driven water evaporation. Nanoscale.

[89] Cheng S., Yu Z., Lin Z., Li L., Mao Z.J.C.E.J. (2020). A lotus leaf like vertical hierarchical solar vapor generator for stable and efficient evaporation of high-salinity brine. Chem. Eng. J..

[90] Soo Joo B., Soo Kim I., Ki Han I., Ko H., Gu Kang J., Kang G. (2022). Plasmonic silicon nanowires for enhanced heat localization and interfacial solar steam generation. Appl. Surf. Sci..

[91] Xiao C., Hasi Q., Wang S., Zhang Y., Li H., Zhang L., Chen L., Li A. (2022). Hollow SiO_2_ microspheres in-situ doped poly(ionicliquid)s gels as efficient solar steam generators for desalination. J. Colloid Interface Sci..

[92] Sun W., Zhong G., Kübel C., Jelle A.A., Qian C., Wang L., Ebrahimi M., Reyes L.M., Helmy A.S., Ozin G.A.J.A.C. (2017). Size-Tunable Photothermal Germanium Nanocrystals. Angew. Chem. Int. Ed..

[93] Gai W., Zhao D.L., Chung T.S. (2019). Thin film nanocomposite hollow fiber membranes comprising Na+-functionalized carbon quantum dots for brackish water desalination. Water Res..

[94] Fathizadeh M., Tien H.N., Khivantsev K., Song Z., Zhou F., Yu M. (2019). Polyamide/nitrogen-doped graphene oxide quantum dots (N-GOQD) thin film nanocomposite reverse osmosis membranes for high flux desalination. Desalination.

[95] Chao W., Li Y., Sun X., Cao G., Wang C., Ho S.-H. (2021). Enhanced wood-derived photothermal evaporation system by in-situ incorporated lignin carbon quantum dots. Chem. Eng. J..

[96] Hamasaki H., Takimoto S., Hirahara K.J.N.L. (2021). Visualization of Thermal Transport within and between Carbon Nanotubes. Nano Letters.

[97] Chen C., Li Y., Song J., Yang Z., Kuang Y., Hitz E., Jia C., Gong A., Jiang F., Zhu J.Y. (2017). Highly Flexible and Efficient Solar Steam Generation Device. Adv. Mater..

[98] Zhu Y., Tian G., Liu Y., Li H., Zhang P., Zhan L., Gao R., Huang C. (2021). Low-Cost, Unsinkable, and Highly Efficient Solar Evaporators Based on Coating MWCNTs on Nonwovens with Unidirectional Water-Transfer. Adv. Sci..

[99] Zhao L., Wang L., Shi J., Hou X., Guo C.F.J.A.N. (2021). Shape-Programmable Interfacial Solar Evaporator with Salt-Precipitation Monitoring Function. ACS Nano.

[100] Zhang P., Liu F., Liao Q., Yao H., Geng H., Cheng H., Li C., Qu L. (2018). A Microstructured Graphene/Poly(N-isopropylacrylamide) Membrane for Intelligent Solar Water Evaporation. Angew. Chem. Int. Ed..

[101] Lu D., Zhou Z., Wang Z., Ho D.T., Sheng G., Chen L., Zhao Y., Li X., Cao L., Schwingenschlögl U. (2022). An Ultrahigh-Flux Nanoporous Graphene Membrane for Sustainable Seawater Desalination using Low-Grade Heat. Adv. Mater..

